# Regional Differences in Infrapatellar Fat Pad Stiffness With Changes in Knee Flexion Angle: A Quantitative Analysis Using Shear Wave Elastography

**DOI:** 10.7759/cureus.101476

**Published:** 2026-01-13

**Authors:** Yoshinori Komatsu

**Affiliations:** 1 Rehabilitation Science, Sendai Seiyo Gakuin College, Sendai, JPN

**Keywords:** infrapatellar fat pad, knee flexion angle, shear wave elastography, tissue stiffness, ultrasound echo

## Abstract

Purpose

This study aimed to quantitatively evaluate regional variations in infrapatellar fat pad (IPFP) stiffness at different knee flexion angles using shear wave elastography in healthy young adults and examined the effects of region and flexion angle.

Methods

Thirty-four healthy young adults participated in this study. IPFP stiffness was measured at four knee flexion angles (0°, 45°, 90°, and 135°) in four regions: subpatellar, central, lateral, and medial. Each condition was measured five times, and the mean value was used for analysis. Two-way repeated measures analysis of variance was performed to assess the effects of knee flexion angle and measurement site.

Results

IPFP stiffness differed significantly by flexion angle (p = 0.010) and region (p = 0.03). A significant interaction between flexion angle and site was also found (p < 0.001). Post-hoc Bonferroni analysis showed no significant pairwise differences among simple main effects.

Conclusion

IPFP stiffness demonstrated angle- and region-dependent patterns in healthy young adults, with significant main effects of knee flexion angle and measurement region as well as their interaction. However, Bonferroni-adjusted post-hoc analyses did not reveal significant pairwise differences between specific conditions. These findings suggest that the IPFP is a structurally complex tissue whose mechanical behavior varies with knee position and interactions with surrounding patellofemoral structures and provide clinically relevant baseline information for physiotherapeutic assessment of knees presenting with anterior knee pain.

## Introduction

The infrapatellar fat pad (IPFP) is a flexible adipose structure located between the joint capsule and synovium, extending from the inferior patella to the anterior tibia. Richly vascularized and innervated, the IPFP contains numerous nociceptors and has been identified as a potential source of anterior knee pain [1,2]. It functions not only as a gliding tissue but also as a sensory organ that perceives mechanical stress and contributes to intra-articular pressure regulation and pain perception.

Clinically, fibrosis, edema, or inflammation of the IPFP has been associated with anterior knee pain [2]. Among patients with osteoarthritis (OA), increased IPFP stiffness correlates with pain severity, independent of Kellgren-Lawrence grade [3]. Similarly, after anterior cruciate ligament (ACL) reconstruction, partial resection, or postoperative fibrosis of the IPFP may reduce elasticity and promote early OA development [4,5]. Understanding changes in IPFP stiffness and mobility is, therefore, essential in physiotherapy; however, clinically practical evaluation methods remain limited.

Previous studies have shown that the IPFP shifts anteroposteriorly during knee movement and undergoes compression or stretching depending on the flexion angle [6]. Although previous studies have reported alterations in IPFP properties under pathological conditions and at specific joint positions, the combined effects of knee flexion angle and regional variation within the IPFP remain insufficiently characterized. Although magnetic resonance imaging can assess IPFP properties, it is costly and time-intensive for clinical application [7]. In contrast, shear wave elastography (SWE) enables noninvasive, rapid, and quantitative evaluation of soft tissue elasticity and is a promising method for visualizing IPFP properties [8]. However, existing SWE studies have often focused on limited measurement sites or single joint positions, making it difficult to interpret how regional stiffness patterns dynamically change across knee flexion angles.

Therefore, the primary aim of this study was to quantify regional IPFP stiffness across multiple knee flexion angles using SWE in healthy young adults and to clarify region- and angle-dependent stiffness characteristics as fundamental baseline data relevant to physiotherapeutic and patellofemoral assessment.

We hypothesized that IPFP stiffness would demonstrate region-specific changes across different knee flexion angles, reflecting distinct mechanical interactions with surrounding patellofemoral structures.

## Materials and methods

Participants

Thirty-four healthy young adults (mean age, 20.2 ± 0.5 years; height, 167.6 ± 7.9 cm; weight, 57.8 ± 8.4 kg) participated. The inclusion criteria were (1) university students aged 18 to 22 years and (2) no history of knee injury or surgery. The exclusion criteria were (1) restricted knee range of motion and (2) current knee pain. Measurements were obtained from the dominant leg, defined as the leg used to kick a ball [9]. The study was approved by the Research Ethics Committee of Sendai Seiyo Gakuin College (approval no.: 0634), and all participants provided written informed consent in accordance with the Declaration of Helsinki.

SWE measurement

All measurements were performed by a single experienced physical therapist on the same day. Participants were positioned supine on a platform. IPFP stiffness was measured using an ultrasound diagnostic system (Aplio 300; Canon Medical Systems, Tokyo, Japan) with a 10-MHz linear probe in SWE mode. Shear wave velocity (m/s) was used as an index of IPFP stiffness [10].

All measurements were performed with participants in a relaxed supine position on a platform. Knee flexion angles were set using a goniometer, and participants were instructed to remain fully relaxed throughout the measurement. Prior to each acquisition, the examiner confirmed the absence of visible quadriceps contraction, and measurements were performed only when the participant was able to maintain a relaxed state without voluntary muscle activation. Knee flexion angles were set at 0°, 45°, 90°, and 135°. Four measurement regions were assessed: subpatellar, central, lateral, and medial. The central region of the IPFP was defined in the longitudinal plane as the midpoint of the patellar tendon, consistent with previous studies [11]. The subpatellar region was assessed using the same longitudinal probe orientation as the central region and was defined as the portion of the IPFP immediately adjacent to the inferior pole of the patella. The medial and lateral regions were defined in the transverse plane at the distal one-third of the patella, in accordance with previous reports [12].

**Figure 1:**
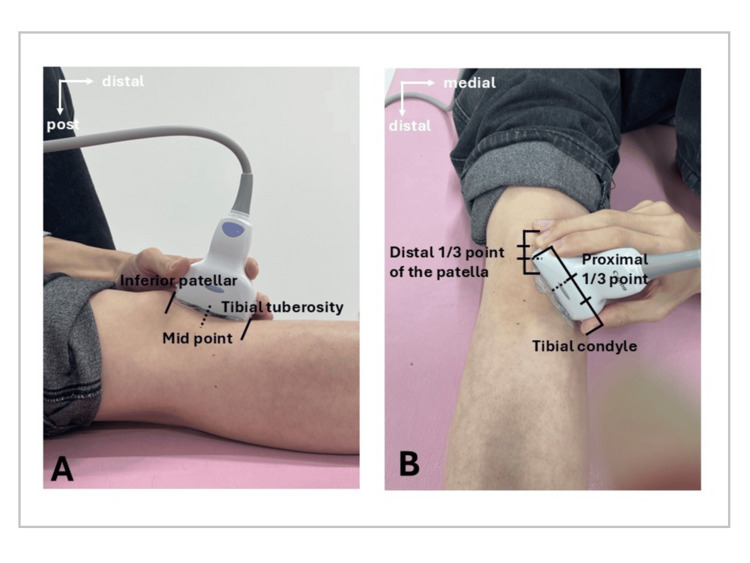
Measurement regions for shear wave elastography of the IPFP (A) Central region: In the longitudinal plane, the central region of the IPFP was defined as the midpoint of the patellar tendon, located halfway between the inferior pole of the patella and the tibial tuberosity. (B) Medial and lateral regions: Measurements were obtained at the proximal one-third point of a line connecting the distal one-third of the patella and the medial or lateral tibial condyle, respectively. IPFP: Infrapatellar fat pad.

Specifically, these regions were determined at the proximal one-third point of a line connecting the distal one-third of the patella and the medial or lateral tibial condyle, respectively (Figure 1). To minimize the influence of probe pressure, the transducer was applied with minimal contact pressure using a generous amount of ultrasound gel, and care was taken to avoid visible tissue compression. Measurements were performed only when a stable and homogeneous shear wave propagation map was visually confirmed. The SWE measurement box was adjusted to include only the IPFP, excluding surrounding tissues. Data with a valid shear wave ratio ≥ 50% were analyzed. To reduce the effects of tissue anisotropy and signal dropout, the probe was carefully aligned perpendicular to the patellar tendon fibers and landmarks maintained in a consistent orientation across all measurements. Trials with evident signal dropout or unstable color maps were excluded and repeated. Each condition was measured five times, and the mean value was used for analysis.

To confirm intra-rater reliability, eight participants were remeasured three times by the same examiner, yielding an intraclass correlation coefficient (ICC [1, k]) of 0.81, indicating good reliability consistent with prior research [3].

Statistical analysis

A two-way repeated measures analysis of variance (ANOVA) was conducted with knee flexion angle (0°, 45°, 90°, and 135°) and site (subpatellar, central, lateral, and medial) as within-subject factors. When Mauchly’s test indicated violation of sphericity, the Greenhouse-Geisser correction was applied. Bonferroni-adjusted post-hoc comparisons were performed where appropriate to identify specific pairwise differences following significant main or interaction effects. The Bonferroni correction was selected to provide a conservative control of type I error associated with multiple comparisons.

Statistical significance was set at p < 0.05. Significant main and interaction effects were interpreted as indicating overall differences or trends across knee flexion angles and measurement sites, whereas Bonferroni-adjusted post-hoc tests were used to examine discrete pairwise differences when applicable. Partial eta squared (η²) and 95% confidence intervals for the main effects were reported to aid interpretation beyond p-values. Partial eta squared (η²) was reported as the effect size. Analyses were performed using SPSS Statistics, version 29.0 (IBM Corp., Armonk, NY).

## Results

Table 1 presents IPFP stiffness values by knee flexion angle and measurement site. Mauchly’s test confirmed sphericity for the angle factor (p = 0.07) but not for the site (p < 0.01) or interaction terms (p < 0.01); therefore, the Greenhouse-Geisser correction was applied.

**Table 1:** Shear wave velocity of the infrapatellar fat pad at each knee flexion angle and region Values are presented as mean ± standard deviation (SD), with 95% confidence intervals (CI) shown in parentheses. Shear wave velocity (m/s) represents the propagation speed of the shear wave measured by shear wave elastography and was used as an index of infrapatellar fat pad stiffness. Two-way repeated measures ANOVA revealed significant main effects of angle (p = 0.010) and site (p = 0.030), as well as a significant angle–site interaction (p < 0.001). Post-hoc Bonferroni comparisons showed no significant differences between any pairs of simple main effects (p > 0.05).

Knee flexion angle	Subpatellar (m/s)	Central (m/s)	Lateral (m/s)	Medial (m/s)
0° (extension)	2.04 ± 0.44 (1.89-2.20)	2.06 ± 0.53 (1.87-2.25)	2.13 ± 0.54 (1.94-2.32)	2.11 ± 0.30 (2.00-2.21)
45° flexion	2.53 ± 1.01 (2.14-2.92)	2.64 ± 1.01 (2.25-3.03)	2.22 ± 0.57 (2.02-2.42)	2.43 ± 0.50 (2.25-2.60)
90° flexion	1.99 ± 0.86 (1.68-2.30)	2.03 ± 0.83 (1.74-2.33)	2.33 ± 0.55 (2.14-2.53)	2.70 ± 0.89 (2.39-3.02)
135° flexion	2.12 ± 0.85 (1.82-2.42)	2.04 ± 0.83 (1.75-2.34)	2.68 ± 0.89 (2.37-3.00)	2.57 ± 0.90 (2.25-2.88)

ANOVA revealed significant main effects of knee angle (p = 0.01) and site (p = 0.03) as well as a significant interaction between the two (p < 0.01) (Table 2). All effect sizes were within the medium range (0.06-0.14) [13]. Bonferroni-adjusted post-hoc tests showed no significant pairwise differences among simple effects (p > 0.05).

**Table 2:** Results of two-way repeated measures ANOVA for shear wave elastography values of the infrapatellar fat pad Values are based on the Greenhouse-Geisser correction when sphericity was violated. df1 and df2 indicate the degrees of freedom for the numerator and denominator, respectively. F represents the F-statistic for the test of main or interaction effects. Partial η² indicates the effect size, representing the proportion of variance explained by each factor. *p < 0.05. ANOVA: Analysis of variance.

Source	df1	df2	F	p	Partial η^2^
Angle	3.00	99.00	3.98	<0.01*	0.11
Site	1.84	60.79	3.86	0.03*	0.11
Angle × Site	6.04	199.40	5.31	<0.01*	0.14

**Figure 2:**
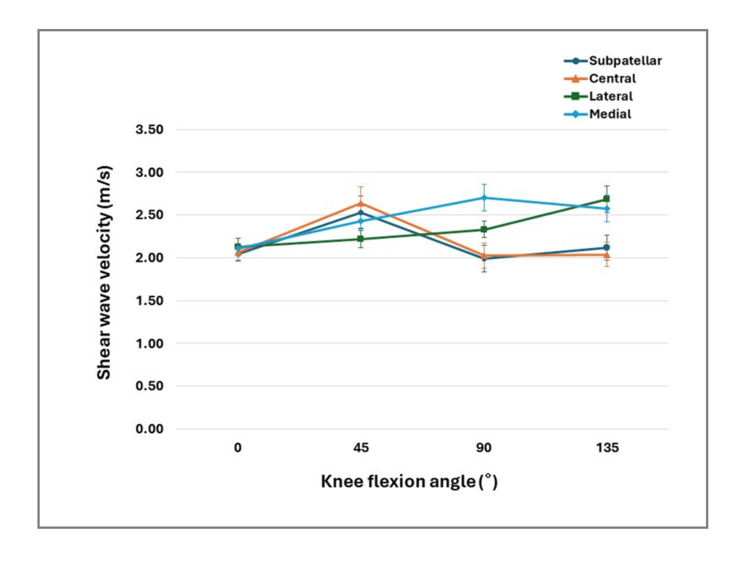
Changes in shear wave elastography values of the infrapatellar fat pad at different knee flexion angles by measurement site Error bars represent standard errors of the mean.

Figure 2 illustrates the changes in shear wave velocity across knee flexion angles for each measurement region. The subpatellar and central regions demonstrated an increase in stiffness at 45°, while the lateral and medial region showed a progressive increase toward 135°.

## Discussion

This study examined the effects of knee flexion angle and measurement site on IPFP stiffness using SWE. Significant main effects and an interaction were identified, although post-hoc comparisons did not reveal pairwise differences.

The observed angle-dependent variation may reflect morphological and mechanical adaptations of the IPFP during knee flexion. As the knee flexes, positional shifts of the patella, patellar tendon, and tibial plateau cause compression, tension, or gliding of the IPFP [6]. Consistent with this study, previous research in patients with OA has shown that IPFP stiffness and elasticity vary with knee flexion angle [14]. In the present study, stiffness tended to be higher at 45° in the subpatellar and central regions, which may be related to compression from the patellar tendon during early flexion. Reports indicate that patellar tendon stiffness is greater at 30° than at 0° or 60° of flexion [15]; thus, increased tendon tension may contribute to localized IPFP stiffening.

In contrast, the lateral and medial regions showed a tendency toward higher stiffness at 135° of knee flexion. During deep flexion, the patella translates laterally, tilts medially, and sinks into the intercondylar groove, increasing patellofemoral contact area and pressure [16,17]. Consequently, the space surrounding the patella narrows, compressing the lateral portions of the IPFP between the femoral condyles and patellar margins. Accordingly, the increased stiffness observed at deep flexion may reflect greater patellofemoral joint contact forces acting on the lateral and medial regions. This interpretation is supported by prior biomechanical and imaging studies reporting increased patellofemoral contact pressures and altered IPFP geometry or mobility with increasing knee flexion [18,19]. Moreover, the medial and lateral patellofemoral retinacula, which extend along the patellar margins, are stretched during knee flexion [20]. The resulting tensile stress likely contributes to the greater stiffness of the lateral and medial IPFP at deep flexion.

The significant main effect of measurement site suggests structural heterogeneity within the IPFP. It is not a uniform adipose mass but a complex tissue interfacing with the patellar tendon, synovium, capsule, and adjacent soft tissues. The subpatellar and central regions may be influenced by their layered architecture and proximity to the patellar tendon, whereas the lateral and medial regions may be affected by patellar tracking and lateral compressive forces. Prior SWE and ultrasonographic studies have reported similar regional differences in IPFP stiffness, with localized increases in stiffness associated with inflammation or fibrosis [3,11].

The significant interaction between knee flexion angle and site suggests that stiffness patterns differ regionally across angles. However, the Bonferroni-adjusted post-hoc comparisons did not identify any significant differences between the pairs of simple main effects. The significant main effects and interaction observed in the two-way ANOVA reflect overall trends in IPFP stiffness across knee flexion angles and measurement sites. The patterns of stiffness change across the measurement sites were generally similar with increasing knee flexion; however, variability within conditions, together with the conservative Bonferroni adjustment, likely limited the detection of statistically significant pairwise differences.

These findings provide physiotherapists with an insight into how knee flexion angle influences regional stiffness of the IPFP, offering clinically relevant baseline information for interpreting knee-related symptoms. In shallower flexion angles, mechanical stress may be greater in the subpatellar and central regions, whereas deeper flexion positions appear to increase mechanical loading on the lateral and medial regions. Such region- and angle-dependent stiffness characteristics may assist physiotherapists in assessing symptoms and in selecting or modifying therapeutic exercises for individuals presenting with discomfort or anterior knee pain during knee flexion activities.

Limitations

The sample size of the present study exceeded that of previous SWE studies investigating IPFP stiffness in healthy individuals, which have reported sample sizes as small as 12 participants [12,21]. Nevertheless, this study included only healthy young adults; therefore, the findings may not generalize to patients with OA or those after ACL reconstruction, who may present with altered IPFP stiffness patterns. Intra-articular pressure and synovial motion were not directly assessed, limiting interpretation of the underlying mechanisms. Inter-rater and inter-device reliability were not examined, and the cross-sectional design prevents causal inference. Additionally, the use of Bonferroni correction, while effective in controlling type I error, is known to be conservative and may reduce statistical power. Therefore, the absence of significant post-hoc pairwise differences should be interpreted cautiously, with consideration of effect sizes and CI.

Although post-hoc pairwise comparisons did not reach significance, these results should be interpreted cautiously. The main effects and interaction demonstrate overall trends rather than discrete differences between specific condition. Bonferroni correction, while conservative, reduces statistical power. The absence of significant differences may be due to limited sample size, moderate effect sizes, and high variability. Moreover, SWE measurements are sensitive to factors such as probe pressure, region of interest placement, tissue depth, and signal validity threshold. Although intra-rater reliability was good (ICC = 0.81), detecting subtle stiffness variations among regions and angles may require larger samples, repeated measurements, or improved measurement precision. Although efforts were made to minimize probe pressure and standardize region of interest placement, SWE measurements remain susceptible to operator-dependent factors, which should be considered when interpreting the absolute stiffness values. Although participants were instructed to remain fully relaxed during measurements and the examiner confirmed the absence of voluntary muscle contraction, quadriceps activation was not objectively monitored using electromyography. As quadriceps tension directly influences patellar tendon stiffness and IPFP compression, residual muscle activity cannot be completely excluded as a potential confounding factor. This methodological limitation should be considered when interpreting the absolute stiffness values and regional differences observed in this study.

## Conclusions

This study quantitatively assessed regional differences in IPFP stiffness across knee flexion angles using SWE in healthy young adults. Significant main effects of knee flexion angle and measurement region, as well as their interaction, were identified. However, Bonferroni-adjusted post-hoc comparisons did not identify statistically significant pairwise differences between the simple main effects. Overall patterns of stiffness change varied regionally with knee flexion, with the subpatellar and central regions tending to show greater stiffness during mild flexion, whereas the lateral and medial regions tended to exhibit increased stiffness during deeper flexion. These findings suggest that the IPFP is a structurally complex tissue whose mechanical properties depend on dynamic interactions with surrounding patellofemoral structures. Understanding these region- and angle-dependent stiffness characteristics provides clinically relevant baseline information that may assist physiotherapists in interpreting knee-related symptoms and in assessing or modifying knee flexion-based exercises. Future studies should investigate regional IPFP stiffness in older individuals and patients with knee pathology, incorporate longitudinal designs, and examine associations with pain, function, and intra-articular biomechanics to clarify the clinical implications of these stiffness patterns.
